# Co-infection of hepatitis E virus and *Plasmodium falciparum* malaria: A genuine risk in sub-Saharan Africa

**DOI:** 10.1186/s13071-021-04723-4

**Published:** 2021-04-20

**Authors:** Syeda Sahra, Abdullah Jahangir, Qasim Zafar Iqbal, Neville Mobarakai, Allison Glaser, Ahmad Jahangir

**Affiliations:** 1 Staten Island University Hospital, Staten Island, NY 10305 USA; 2 King Edward Medical University, Lahore, 54000 Pakistan; 3 Department of Internal Medicine, Hofstra School of Medicine, Staten Island University Hospital, 475 Seaview Avenue, Staten Island, NY 10305 USA

**Keywords:** COVID-19, Cerebral malaria, Hepatitis E virus, HEV, *Plasmodium falciparum*

## Abstract

**Background:**

There is a high prevalence of malaria and viral hepatitis in South Africa. Co-infection with *Plasmodium* malaria (leading to cerebral malaria) and hepatitis E virus (HEV) is a rare phenomenon.

**Case presentation:**

A 33-year-old African American male with no past medical history developed altered mental status on his return from Ivory Coast. His blood tests were significant for renal and liver failure and a high *Plasmodium* parasite burden of 33% on the blood smear. Interestingly, he also had a positive result for hepatitis E IgM. The patient was effectively treated with aggressive hydration and intravenous (IV) artesunate.

**Conclusion:**

Our report is the first to our knowledge in the cerebral malaria literature on a patient with hepatitis E co-infection. This exciting case emphasizes the importance of considering all kinds of endemic infectious diseases when evaluating sick returning travelers presenting to the emergency department.

## Introduction

*Plasmodium* malaria, a mosquito-borne parasitic disease, has been known to humankind since early times. It presents mainly as high-grade fevers, chills, nausea, gastrointestinal symptoms, and anemia. Its high morbidity and mortality are significant health concerns for third world countries even in the current times of advanced treatment and prophylactic options.

Cerebral malaria is one of the deadliest manifestations of *Plasmodium falciparum* infection. Cerebral malaria presents mainly as a neurological sequela secondary to microvascular inflammation from sequestered blood cells, interaction between adhesive proteins and endothelial surfaces, generation of cytokines, and disruption of the blood-brain barrier [1, 2]. Altered mental status is further compounded by accompanying hypoglycemia and electrolyte abnormalities from multiorgan failure.

The prevalence and severity of cerebral malaria are higher in children with life-long neurological complications [3]. Pediatric age group presentation encompasses mainly coma and seizure due to cerebral edema [4]. A study by Carter et al. suggests that cerebral malaria may be responsible for most acquired language disorders [5]. A similar inverse association between cerebral malaria and childhood cognition disorders was established by Boivin et al. [6].

In adults, multiorgan failure usually accompanies the state of altered mental status and coma. Furthermore, neurological deficits are generally long lasting in adults. The treatment of choice for cerebral malaria is intravenous artesunate, including the pediatric and pregnant subgroups [7].

Sub-Saharan Africa is the hub of several infections due to the low socioeconomic status, lack of hygiene and sanitation measures, and inadequate health resources. Hence, both the incidence and prevalence of malaria, human immunodeficiency virus (HIV), and hepatitis are exceedingly high in this region, accounting for the high regional morbidity and mortality. Recent studies have been done to document and understand the co-infection trends of hepatitis B, hepatitis C, and HIV in the Ivory Coast [8]. Interestingly, there is a high incidence of malaria and hepatitis E virus (HEV) infection in the sub-Saharan population [9]. HEV, spreading through the oral-fecal route, is known to resolve with symptomatic management in most cases except in pregnant females and individuals with hepatic insufficiency. Unfortunately, the clinical course can escalate quickly to fulminant liver failure if there is a co-infection with a malarial parasite. On literature review, rare cases of co-infection with *Plasmodium* and HEV have been reported with an abysmal prognosis. For interest and awareness, we report an unusual case of a co-infection of hepatitis E in a patient who developed cerebral malaria from *Plasmodium falciparum* infection [10].

## Case presentation

A 33-year-old African American man with no past medical history was evaluated in the emergency room (ER) 3 weeks after returning from Ivory Coast. He was born in Ivory Coast and came to the USA as a toddler. Since then, he had been visiting Abidjan, Ivory Coast, every few years to see family. Recently, he spent 1 uneventful month in Abidjan and returned to the USA. He denied any history of malaria in the past. He did not take any anti-malarial prophylaxis during the recent visit. He confessed to smoking a few tobacco cigarettes daily but denied any illicit drug abuse. He developed a fever after 7 days. The fever was high grade (103 °F) in intensity and intermittent, with each episode lasting several hours. The patient took multiple tablets of over-the-counter Tylenol and Motrin for the fever at home without any significant improvement.

The patient had visited the ER twice in the last week for a similar complaint of low-grade fevers. Thrombocytopenia and transaminitis were seen on blood testing. However, given the COVID-19 pandemic, recent international travel, and blood testing results bearing similarity to COVID-19, he was promptly tested for COVID-19. He was discharged home after the COVID-19 polymerase chain reaction (PCR) test result was negative. The fevers persisted at home for the next 3 days, nonetheless. Two days before the presentation, he developed chills, nausea, and vomiting. The vomiting was initially bilious and later became dark-colored. The patient's family noticed that his mental status had gradually worsened over the past week, and they decided to bring him to the ER again.

On this visit to the ER, he had an initial temperature of 101.4 °F, blood pressure of 122/78 mmHg, respiratory rate of 18 breaths per minute, and oxygen saturation of 98%. Physical examination revealed scleral icterus. The patient was oriented only to himself with a Glasgow coma scale (GCS) score of 13 out of 15. The physical examination did not reveal any hepatosplenomegaly, flapping tremors, or focal neurological deficits.

The initial blood testing was significant for hypoglycemia, pancytopenia (microcytic anemia and thrombocytopenia), elevated lactate level, and transaminitis (AST 75 U/l, ALT 36 U/l, bilirubin 15.2 mg/dl, alkaline phosphate 124 U/l) (Tables 1 and 2). The COVID-19 PCR was negative again.

**Table 1:** Hematology and comprehensive metabolic panel studies throughout hospitalization and discharge

Hematology and comprehensive metabolic panel studies	Day 1	Day 2 am	Day 2 Pm	Day 3	Day 4	Day 6 (discharge)	Ten days after discharge
Hemoglobin g/dl	14	11.5	10	8.6	8.1	7.4	8.2
WBC count k/ul	7.75	9.2	10.7	12.8	12.4	16.3	8.9
Platelet count u/l	36	94	76	91	158	306	541
Lactate mmol/l	8.4		2.6				
AST U/l	75		116	229	284	193	50
ALT U/l	36		48	45	91	156	95
Alkaline phosphate U/l	124		116	83	104	157	
Albumin g/d/l	3.6		2.8	2.6	2.5	2.6	4.2
Gamma-glutamyl transferase U/l			45				
Bilirubin total mg/dl	15.2		18.4	21.6	22.5	7.9	3.2
Parasitemia %age	33		31.4	One negative smear	0.02%	0.02%	
BUN mg/dl	34		28	26	26	14	17
Creatinine mg/dl	1.4		1.4	1.6	1.8	1.7	< 0.5

**Table 2:** Hemolysis panel

Ferritin ng/ml	6853
Ceruloplasmin mg/dl	44
Transferrin mg/dl	161
Lactate dehydrogenase U/l	1102
Haptoglobin mg/dl	< 20

**Table 3:** Immunology panel

Cryptococcal antigen	Negative
West Nile virus by PCR	Negative
Leptospirosis antibodies	Negative
Hepatitis A IgM	No
Hepatitis A IgG	Reactive
Hepatitis E Ab IgM	Reactive
Hepatitis C virus cut-off index	0.04
Hepatitis E IgG	Negative
Hep B surface antigen	Negative

**Fig. 1:**
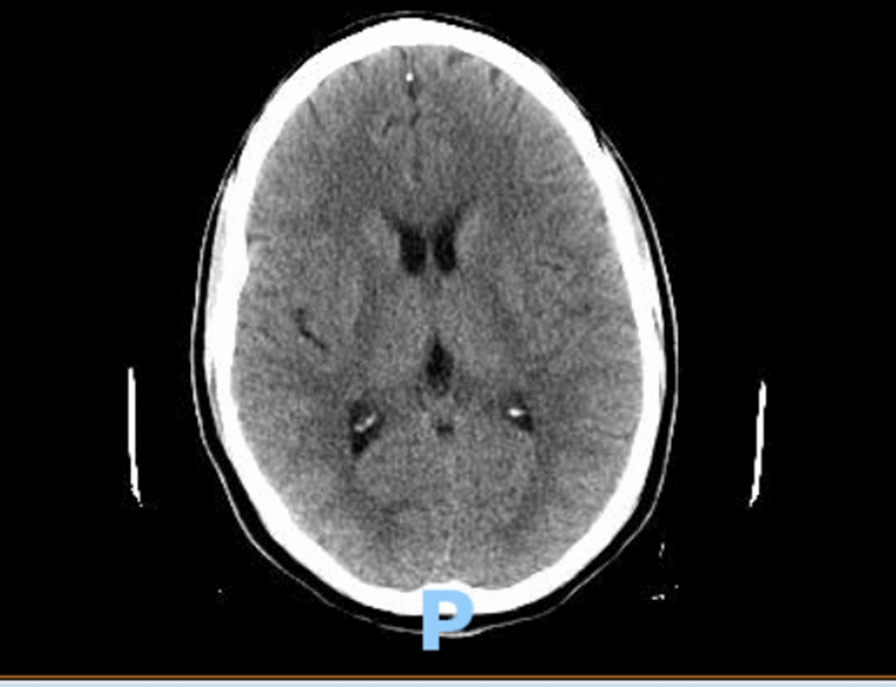
CT head without any evidence of intracranial changes, cerebral edema, mass effect, or midline changes

This time the medical team consulted the Infectious Diseases service, which recommended obtaining an immediate peripheral blood smear and acute hepatitis panel. A peripheral blood smear showed intraerythrocytic parasites consistent with *Plasmodium falciparum* with a high degree of parasitemia (33%). A lumbar puncture was recommended to rule out cerebral malaria as the patient was confused. The blood alcohol, acetaminophen, and salicylate levels were normal. Hepatitis E IgM was positive, and the rest of the acute hepatitis panel was negative (Table 3). There was no evidence of retinopathy on fundoscopic examination. A diagnostic lumber puncture was performed to rule out cerebral infection. The initial cerebrospinal fluid (CSF) studies were unremarkable for any elevated cell count, glucose, or proteins. CSF PCR studies for *E. coli, H. influenzae, Listeria monocytogenes, Neisseria meningitidis, Streptococcus agalactiae, Streptococcus pneumoniae*, sytomegalovirus (CMV), *Cryptococcus neoforms*, herpes simplex virus (HSV-1, HSV-2), human herpesvirus 6 enterovirus, parechovirus and varicella zoster virus (VZV) came out negative (Tables 4 and 5). The head CT scan checked for any bleeding, mass effect, or midline shift (Fig. 1).

**Table 4:** CSF studies

Color	Xanthochromia
Glucose mg/dl	62
Protein mg/dl	40
RBC count	2
Total nucleated cell count	2
VDRL titer	Nonreactive
LDH, CSF (u/l)	30

**Table 5:** CSF PCR studies

West Nile IgM	Negative
West Nile IgG	Negative
West Nile virus by PCR	Not detected
CSF cultures	No growth
HSV ½ PCR	Negative
E. coli K1	Negative
*Streptococcus agalactiae*	Negative
*Streptococcus pneumoniae*	Negative
Cytomegalovirus	Negative
Human herpes virus 6	Negative
Human parechovirus	Negative
Cryptococcus neoformans/gattii	Negative
Lyme PCR, result	Negative
*Haemophilus influenzae*	Negative
*Listeria monocytogenes*	Negative
*Neisseria meningitidis*	Negative
Human parechovirus	Negative
*Plasmodium*, CSF PCR	Positive

Given the high degree of parasitemia and concern about cerebral malaria, we consulted the regional Center for Disease Control and Prevention (CDC). The patient was started on intravenous artesunate (2.4 mg/kg at 0 h, 12 h, and 24 h). A dramatic improvement was seen in his blood work and mental status from hospital day 3. He was fully alert, awake, and oriented to himself, time, and place by hospital day 5. Parasitemia was also entirely resolved by hospital day 5 (0.02%). The liver enzymes trended down, and the platelet count was back to normal (Tables 1 and 2). Three negative blood smears were obtained, and the patient was discharged home on Coartem (20 mg/120 mg tablets, four tablets as the initial dose, four tablets again after 8 h, and then four tablets twice daily for the following 2 days) with instructions to be closely monitored by the primary care physician. The medicine team followed the patient on telehealth, and he reported doing well and performing his daily activities without any restrictions. CSF PCR for *Plasmodium* tested positive 3 weeks later, confirming the diagnosis of cerebral malaria. His liver enzymes and bilirubin further trended down on repeat outpatient blood testing after discharge from the hospital (Table 1).

## Discussion

Malaria has a high mortality rate, particularly in the African pediatric population, claiming approximately half a million lives each year [11]. Ninety-two percent of global malarial cases were reported in the World Health Organization (WHO) African Region alone in 2017. Fatal complications, including cerebral malaria, acute respiratory distress syndrome (ARDS), and renal failure, can arise. Severe malaria was attributed to approximately one-tenth of the hospital mortalities in a hospital study done in Abidjan [12].

Apart from mortality, cerebral malaria is a major risk factor for long-term neurological sequela, including but not limited to epilepsy [13]. Fundoscopy has been employed more frequently recently to evaluate cerebral circulation in patients suspected to have cerebral malaria. We can see patchy retinal whitening and vasculature color changes, pointing toward cerebral microvasculature. The anemia in patients with malaria can be prolonged, though, and artesunate can be a contributing factor.

Our case is unusual as the patient had a co-infection with HEV along with malaria. The transaminitis and his return from Africa triggered the testing for an acute hepatitis panel on the Infectious Diseases team's recommendation. The rest of the hepatitis panel was negative. The patient reported no gastrointestinal symptoms, which is one of the common manifestations of HEV. IgM for hepatitis E was positive, which indicated an acute infection. IgM is positive after the incubation period for HEV is completed, which can range from 2 to 6 weeks. This is followed by a positive IgG value within a very few days. This incubation period and timing for positive IgM and negative IgG at the time of hospitalization confirmed an acute HEV. Workup ruled out other viral causes of transaminitis.

Similarly, the incubation period of *Plasmodium falciparum* malaria ranges from a week to a month, which can explain the delayed presentation after the return and peak of symptoms (confused mental status, fever, transaminitis) after approximately 3 weeks. The CSF PCR was positive for *Plasmodium falciparum*, which established a cerebral malaria diagnosis. There was no evidence of cerebral edema, mass effect, or midline shift on CT head. The fundoscopic examination was unremarkable. The response to artesunate was rapid with prompt resolution of parasitemia on the peripheral smear. The only limitation in this study was the inability to repeat hepatitis E IgG analysis on outpatient blood testing as it would have left no stone unturned in confirming the diagnosis of acute HEV based on the projected timeline. Unfortunately, we were limited in our testing by financial constraints of the patient.

The two infections (HEV and malaria) have different vectors and different mechanisms of causing harm to human hosts. HEV spreads through the fecal-oral route. The female *Anopheles* mosquito transmits *Plasmodium* into human blood, which reaches the liver, undergoes asexual reproduction, and completes development prior to infecting red blood cells and multiplying [14]. The consequent disease manifestations are the result of microvascular occlusions and inflammatory responses generated by infected and ruptured erythrocytes.

The initial clinical presentation of altered mental status can be very misleading for the physician and unfortunate for the patient. Fever, thrombocytopenia, and abnormal liver enzymes paint a clinical picture remarkably familiar to COVID-19 in the current circumstances heightened by recent travel [15]. Delay in diagnosis and treatment of cerebral malaria is undisputedly terminal. Cerebral malaria has been misdiagnosed as psychosis and human rabies in the past [16]. Likewise, the absence of pharmacological prophylaxis or incorrect dosage puts a person at exceedingly high risk of cerebral malaria and its mortality [17–19].

While reviewing the literature, a study done in Afghanistan demonstrated a high incidence of hepatitis A and E and malaria due to a lack of health resources and sanitation measures [20]. Barcus et al. studied the association of hepatitis B and malaria in an endemic population in Vietnam, showing a higher tendency of patients in subgroups with cerebral malaria to test positive for HbsAg [21].

Furthermore, co-infection with HEV and malaria has been reported with fatal consequences [22, 23]. So far, this is one of the first cases of co-infection where a patient's malaria had advanced to cerebral malaria and the patient still had a favorable outcome. According to a WHO report (2018), malaria's mortality rate is remarkably higher in females and younger children. This population is at higher risk of morbidity and mortality from HEV as well. The immune response is diminished with severe malarial infection, which can predispose patients to greater damage from HEV.

Potential synergistic hepatotoxicity was suggested by Nasir et al. when they studied hepatitis C and malaria co-infection [19]. Analogous co-infection in a person with underlying hepatic insufficiency or a pregnant woman can indeed be fatal and should be kept in mind. HEV causes viral cytotoxicity, while *Plasmodium* malaria affects the hepatic microvasculature by endothelial injury from the parasitized erythrocytes. A case of sporadic HEV in a patient with a history of malaria has been reported in the USA before, and physicians should be reminded of this association in relevant clinical settings even in first world countries [24]. Clinicians can avoid many neurological side effects if cerebral malaria is diagnosed in a timely manner and treatment is initiated [25, 26].

## Conclusion

Malaria and viral hepatitis are indeed not the first thought in a physician's mind when a patient comes in with vague complaints in an emergency room in a first world country. Particularly in the current circumstances, the threat of COVID-19 infection is real and is one of the top differential diagnoses in travelers. The liver enzyme levels in these patients are usually elevated, and suspicious personnel is managed conservatively with quarantine even if they test negative for COVID-19. COVID-19 should not hinder the thought process from ruling out other infectious etiologies. The suspicion of malaria and hepatitis should be high in patients with a recent travel history to endemic areas, particularly South Africa and South Asia. There is an exceedingly high mortality rate for malaria, particularly cerebral malaria, and efforts should be made to achieve prompt diagnosis and treatment. A detailed history should be taken from all available sources, and relevant blood testing should be ordered. Transaminitis and hyperbilirubinemia should raise the suspicion of potential endemic infections, such as malaria and viral hepatitis [27]. We propose that transaminitis should always trigger testing for peripheral blood smears and an acute hepatitis panel in sick returning travelers. Physicians should be aware of unconventional presentations with co-infection from common pathogens to avoid a delay in diagnosis. The already burdened health care systems cannot afford missed diagnoses of co-infections. A delay in a similar case of cerebral malaria and acute viral hepatitis can lead to fulminant hepatic necrosis with possibly fatal consequences. Lastly, timely detection and treatment saved this lucky patient from any neurological deficits, which is always a potential complication of cerebral malaria as discussed above. These patients should be closely monitored as outpatients after discharge. Travelers should utilize anti-malarial prophylactic medications and precautionary measures, including the use of long-sleeve clothing, mosquito repellents, and mosquito nets. Hepatitis A and E viruses mainly spread through the fecal-oral route. Clinicians should offer hepatitis (A, B, E) vaccinations to the patients and their close contacts to prevent illness in future infection events.

## Data Availability

Patient-specific data were obtained from hospital electronic medical records of Northwell Health, and patient identifying information was cropped.

## Abbreviations

E.R.: Emergency room
IV: Intravenous
ARDS: Acute respiratory distress syndrome
AST: Aspartate aminotransferase
ALT: Alanine aminotransferase
CDC: Centers for Disease Control and Prevention
CSF: Cerebrospinal fluid
GCS: Glasgow coma scale
PCR: Polymerase chain reaction
